# Two-color pump-probe interferometry of ultra-fast light-matter interaction

**DOI:** 10.1038/s41598-017-10709-z

**Published:** 2017-09-04

**Authors:** Yoshio Hayasaki, Shin-ichi Fukuda, Satoshi Hasegawa, Saulius Juodkazis

**Affiliations:** 1 Center for Optical Research and Education (CORE), Utsunomiya University, 7-1-2 Yoto, Utsunomiya, 321-8585 Japan; 2 Centre for Micro-Photonics, Swinburne University of Technology, Hawthorn, VIC 3122 Australia; 3 Center of Nanotechnology, King Abdulaziz University, Jeddah, 21589 Saudi Arabia; 4 Melbourne Center for Nanofabrication, Australian National Fabrication Facility, Clayton, VIC 3168 Australia

## Abstract

Two-color side-view probing of light-matter interaction from minute focal volume of a tightly focused fs-laser pump pulse reveals charge dynamics with high 0.9 *μ*m optical resolution and approximately ~45fs temporal resolution defined by pulse duration. Use of two colors is advantageous for probing optically excited plasma regions with different density. Holographical digital focusing and spatial filtering were implemented to obtain the same resolution images for subsequent Fourier analysis. Fast plasma density decay with time constant ~150 fs was resolved and is consistent with self-trapping. Potential applications of an optical control over light-induced defects with deep-sub-wavelength resolution is discussed.

## Introduction

Imaging and detection of ultra-fast phenomena is in constant development using different methods of pump-probe techniques at different magnifications and resolutions^1–6^. High spatial resolution of a pump-probe optical imaging of tightly focused femtosecond (fs)-laser pulses inside glass carried out in lateral^7^ and axial^8^ views with interferometric technique was ~0.2, 0.6 *μ*m, respectively. The side-view (axial) imaging is sought after due to possibility to use shorter wavelength strobe (probe) pulses and to achieve high resolution even at moderate focusing with objective lens of numerical aperture $NA\simeq 0.5$. Formation of plasma filaments, self-trapped excitons, shock waves, axially extended voids^9^, tubular compressed micro-volumes^10^ and bulk ripples^11^ can be potentially resolved using such side-view imaging. Those phenomena are highly dynamic and have transient stages which are not observed in standard *post mortem* inspection of the samples or are detected with a time integration causing loss of  temporal resolution^12–15^. Pump-probe methods are fast evolving to improve spatial and temporal resolutions, e.g., tilted fs-laser pulses were used to increase region of spatial-temporal overlap in imaging of filaments in water^3^, to reveal the mechanism of water ionisation^16^, digital holography was applied to reach high temporal resolution of filamentation and ablation of transparent dielectrics^17^, the Abel inversion was used to reconstruct the temporal density evolution of plasma in air^18^.

The current state of the art in generation of ultra-short laser pulses is at sub-100-as^19^, however, those pulses have photons of tens-of-eV energy and are less useful to probe optically excited plasma inside transparent materials. Ultra-short Fourier transform limited *t*_*p*_ = 45 fs pulses of *λ* = 800 nm wavelength have a spectral width of ${\rm{\Delta }}\lambda ={\lambda }^{2}/(c{t}_{p})\simeq 47$ nm and are well suited to probe inner regions in dielectrics with strongly excited micro-plasma or undergoing phase transitions. The phase transitions is an active research focus, in particular, a non-thermal melting^20^ is debated which is deemed improbable by thermodynamics arguments^21^. A spatial resolution down to ~10 nm would be required to reveal polymerisation enhancement along the direction of polarisation of tightly focussed fs-laser pulses in a direct laser writing^22^. Temporal resolution of chemical reactions and molecular ionisation have been recently demonstrated with sub-10 fs resolution^23^. Hence, probing a light-matter interaction with an increasing spatio-temporal resolution is a continuing quest.

Here, a novel interferometric pump two-color probe method is developed for a high spatial 0.9 *μ*m and temporal 45 fs imaging of ultra-fast light-matter interaction in the bulk of transparent sample. Combination of two-color probes allows to better visualise plasma excited by the pump pulse due to difference in absorbance at two different wavelengths.

## Experimental

### Optical setup

**Figure 1:**
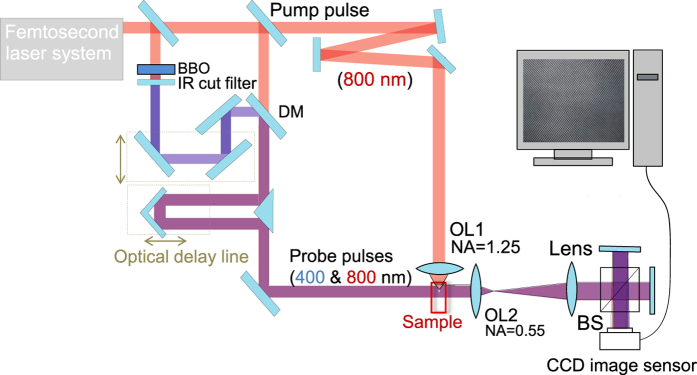
Setup of the pump and two color probe interferometry. OL1, 2 are the objective lenses with corresponding numerical apertures NA, BS is the beam splitter, DM - dichroic mirror to combine 800 and 400 nm pulses, BBO - *β*BaBO_4_ crystal is for frequency doubling (*λ* = 400 nm wavelength).

Figure 1 shows an experimental setup of the pump-probe interference microscope with two-color probes. The pump pulse with a center wavelength of *λ*_*ω*_ = 800 nm generated by an amplified fs-laser system was focused inside a glass sample from its sidewall using a 100^×^ oil-immersion objective lens (OL) with a numerical aperture *NA* of 1.25. The sample was a superwhite crown glass (B270, Schott), which is representative of the widely used low dispersion glasses. The laser-induced phenomena was created by a single pulse irradiation and the sample was moved transversely every one pulse. Focus was placed only 10 *μ*m below the surface to minimize spherical aberration.

The two-color probes are the fundamental wavelength of *λ*_*ω*_ = 800 nm and its second-harmonics with a center wavelength of *λ*_2*ω*_ = 400 nm. The second-harmonics probe pulse was generated in a barium borate (BBO) crystal. Both two-color probe pulses were irradiated onto the sample with a delay time *T* after arrival of the pump pulse and was introduced to the interferometer after passing through the sample using a telecentric microscope optics composed of an 50^×^ OL with a focal length of 4.0 mm and *NA* of 0.55 and a doublet lens with a focal length of 500 mm.

Control of the pump pulse duration was made by an *in situ* monitoring the breakdown inside the glass and tuning an outside-cavity compressor: the lowest pulse duration at which the optical breakdown was observed was also causing the fastest change of the phase due to Kerr effect at the focal spot. The pulse duration at the laser output was 45 fs and the Kerr changes of the signal were comparable. This set the temporal resolution of experiments. The duration of the probe pulse was minimized by monitoring the most efficient SHG while pre-chirping pulse at the fundamental frequency.

The delay *T* was controlled with an optical delay line with the maximum delay of 10 ns. The delay line was composed of two stepping-motor-driven stages for controlling the fine and coarse movements. The fine stage for the delay less than 1 ps had a step movement 0.5 *μ*m, a resolution of 3 *μ*m at the maximum translation of 25 mm. The coarse stage for delays longer than 2 ps had a translation step of 25 *μ*m, resolution of 16 *μ*m, and the stroke length of 1500 mm.

The coincidence *T* = 0 ps time between pump and probe was decided when there was no recognizable photomodification at the pump pulse of ~50 nJ. Precision of the time reference frame was comparable with the pulse duration, which allowed to resolve Kerr changes in the phase images in pump-probe experiment. In this study we focussed on plasma formation before the void formation which was observed for the pump pulse energies above 300 nJ at the same focusing conditions as was reported previously^8^.

## Image Analysis

Interference fringes were recorded by the angularly multiplexed method with a cooled charge-coupled device (CCD) image sensor (BU-50LN, Bitran) with a pixel size of 8.3 *μ*m and 16-bit recording, and were read out by a computer for analysis. The angularly multiplexed method is described later in detail elsewhere^1^.

The plasma emission was observed at the focus point, however, the intensity was not so high in the used glass. The integrated intensity of plasma emission was < 20% as compared with intensity of the probe beam with the image sensor detection. The plasma emission superimposed on the interference images was subtracted from the raw interference image before numerical procedures.

**Figure 2:**
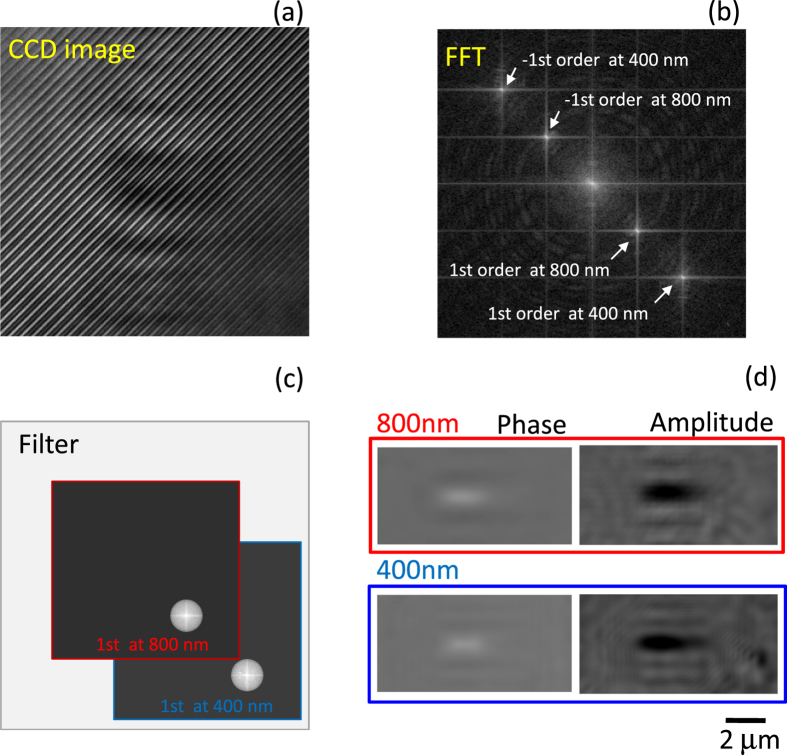
Implementation of the method. A CCD image (**a**) and its Fourier transform (**b**) with selection of the filter apertures for back transform (**c**) with the resulting phase and amplitude images (**d**). The filters (in (**c**)) are set to the optical resolution of setup at 0.89 *μ*m.

Complex amplitude of the interference image was obtained by the Fourier transform method^24^. More detailed procedure is described below. Let us consider an interference between an one-dimensional object wave with an amplitude *a*_*s*_(*x*) and a wave number *k*_*s*_(*x*) with a plane reference wave with an amplitude *a*_*r*_ with a wave number *k*_*r*_. It is supposed that *k*_*s*_(*x*) has the object signal components *k*_*s*1_(*x*) around the center *k*_*s*0_. The fringes for each wavelength is described as:

1 $$I(x)={a}_{r}^{2}+{a}_{s}{(x)}^{2}+{a}_{r}{a}_{s}(x)\exp \,i[-Kx-\varphi (x)]+{a}_{r}{a}_{s}(x)\exp \,i[Kx+\varphi (x)],$$

where $K={k}_{s0}-{k}_{r}$ and $\varphi (x)={k}_{sl}(x)x$. The wave number of the fringes at 400 nm, denoted as *K*_2_, is twice of that for 800 nm, denoted as *K*_1_, that is *K*_2_ = 2*K*_1_. The Fourier transform of the superposition of the fringes formed by two wavelengths described as:

2 $$F[\sum _{n\mathrm{=1,2}}{I}_{n}(x)]=\sum _{n\mathrm{=1,2}}{A}_{n}(\omega )+{B}_{n}{(\omega +{K}_{n})}^{\ast }+{B}_{n}(\omega -{K}_{n}),$$

where *F* is the Fourier transform, *n* = 1 and *n* = 2 are the values of 800 nm and 400 nm, respectively, ${A}_{n}(\omega )=F[{a}_{n,r}^{2}+{a}_{n,s}^{2}(x)]$ and ${B}_{n}(\omega -K)=F[{a}_{n,r}{a}_{n,s}(x)\exp (i\varphi (x))\exp (i{K}_{n}x)]$, and * marks the complex conjugate. If $max[A(\omega )]+max[{B}_{1}(\omega )] < {K}_{1}$ and $max[{B}_{1}(\omega )]+max[{B}_{2}(\omega )] < {K}_{1}$, that is, the spectra of *A*(*ω*), *B*_1_(*ω*), and *B*_2_(*ω*) have no overlap over each other, they can be separated by a spatial frequency filter on the Fourier plane as shown in Fig. 2(b). The filter *W*(*ω*) used in the experiments was of the Hanning type, defined by:

3 $$W(\omega )=\{\begin{array}{ll}\mathrm{[1}+\,\cos (|\omega -{K}_{n}|\mathrm{/2}h\mathrm{)]/2,} & {\rm{if}}\,|\omega -{K}_{n}|\le 2\pi h\\ \mathrm{0,} & {\rm{otherwise}}\end{array}$$

where *h* is the filter width.

Resolutions of the interference observation were 0.44 *μ*m at 400 nm and 0.89 *μ*m at 800 nm, which were calculated from a full width at half maximum (FWHM) of the Airy disk pattern using the *NA* = 0.55 objective lens; the magnification of the microscope, *M* = 125 and the cut-off spatial frequency of the Fourier filtering *h* (described in the next section) was 12 line pairs per millimeter (lp/mm).

In the framework of geometrical optics and with assumption of aplanatic focusing, a computed cut-off frequency of the total system was *Mh* = 1470 lp/mm and the corresponding resolution limit was 1/*Mh* = 0.68*μ*m. This is larger than the resolution of 0.44 *μ*m at 400 nm and smaller than the resolution of 0.89 *μ*m at 800 nm. In order to equalize both resolutions, the cut-off frequency of the filter for the 400 nm images was decreased. After both *B*_*λ*_(*ω* − *K*) were extracted by the spatial frequency filter, the complex amplitudes were obtained by the inverse Fourier transform as:

4 $$b(x)={a}_{r}{a}_{s}(x){e}^{i\varphi (x)}{e}^{iKx}\mathrm{.}$$

Each measurement had two image captures. First, image was captured just before the pump-pulse irradiation to obtain the reference. Second, the image was taken with pump present and the ratio was calculated. The used procedure was tested experimentally to have a negligible effect of crosstalk between the two filters at 400 nm and 800 nm. However, for more complex images with high content of high spatial frequencies the cross talk might occur. It could still be eliminated by selection of a slightly different angles of incidence for the two probes.

In order to eliminate the tilt component in the output images, they were normalized by the output images obtained previously without an object, described as ${b}_{0}(x)={a}_{r}{a}_{i}{e}^{iKx}$, where *a*_*i*_ is the amplitude of the illumination light. Finally, the normalized output images were obtained as

5 $$\tilde{b}(x)=\frac{{a}_{s}(x)}{{a}_{i}}{e}^{i\varphi (x)},$$

where ${a}_{s}(x)/{a}_{i}$ is the transmittance of the object.

The complex amplitudes at 400 nm and 800 nm were simultaneously measured using the same optical system. The wide difference of wavelengths gave a color aberration and the best focusing position of a lens were different. The deviation was corrected by the digital focusing using the diffraction calculation based on the angular spectrum method^25^. This digital focusing is one of the advantageous features of the used digital holography approach.

## Results

In previous study, a side-view imaging at a single 400 nm wavelength allowed to see formation stages of strong refractive index modifications, associated with plasma density dynamics, at the earlier times, followed by shock wave formation, molten flow of material, and void formation when imaging was carried out till delays of few nanoseconds^8^. Here we focus on the early stages of plasma formation at smaller excitations.

**Figure 3:**
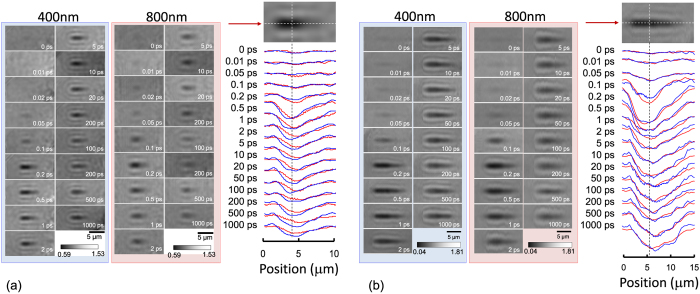
Amplitude of FFT image at 400 nm and 800 nm wavelengths after excitation pump pulse of *E*_*p*_ = 50 nJ at focus (**a**) and 200 nJ (**b**) excitation together with the on-axis cross sections along pulse propagation; the amplitude scale was the same for both wavelengths. Measurements at both wavelengths was carried out simultaneously.

Figure 3 shows the amplitude of FFT at two 800 and 400 nm wavelengths and its axial distribution along the propagation of the pulse. The amplitude changes are related to the absorptivity of a plasma at the focal region depending on the energy of the pump pulse. Slightly stronger amplitude changes observed at 400 nm wavelengths for the 50 nJ pulse (Fig. 3(a)) were related to a better transmission of the short wavelength pulses through the plasma region. For the 200 nJ pulse, when a high critical plasma density is reached even for the shorter wavelength (a plasma screening), the changes of amplitudes at both wavelengths were comparable (Fig. 3(b)). Differences of transmission (and diffraction) through (and around) the plasma in focal region at two wavelengths was further scrutinized using the ratio of transmissivity discussed next.

**Figure 4:**
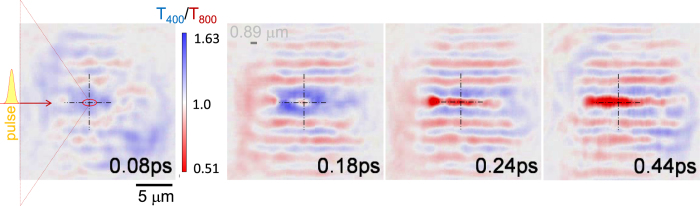
Transmission ratios at 400 nm and 800 nm, *T*_400_/*T*_800_, at different times after single pulse of *E*_*p*_ = 200 nJ focused by $NA\equiv n\,{\rm{s}}{\rm{i}}{\rm{n}}\,\alpha =1.25$ (*α* = 55.6°) objective lens (shown by a triangle); the optical resolution of the FFT filter was 0.89 *μ*m marked by ellipse. Fringing is caused by division of two images with similar intensity values and the FFT enhanced  oscillations.

**Figure 5:**
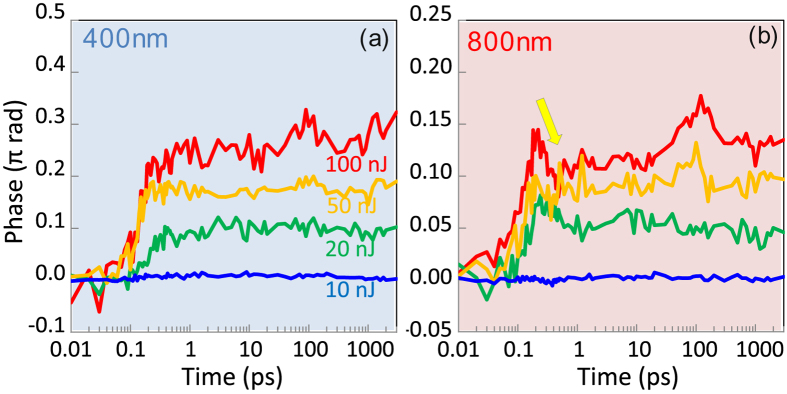
Phase of FFT images at 400 nm (**a**) and 800 nm (**b**) wavelengths at different pump pulse energies from *E*_*p*_ = 10 nJ to 100 nJ. An arrow shows a characteristic transmission decrease at the end of laser pulse. Positive phase corresponds to the −Δ*n* change of refractive index, and vice versa.

In this new two-color probing, it is informative to present images as a ratio of the transmitted light at two wavelengths, *T*_400/_ *T*_800_ (Fig. 4). Transmission at 400 nm has a larger cut-off (critical) electron density ${N}_{c}={\varepsilon }_{0}{m}^{\ast }{(\frac{2\pi c}{e\lambda })}^{2}$, where m*, *e* are the optical effective mass and charge of electron, respectively, *ε*_0_ is the permittivity of vacuum, *c* is the speed of light. The blue-color probe (400 nm) can see through the focal region until the electron plasma density becomes ${N}_{c}^{\mathrm{400\ }{\rm{nm}}}\simeq 6.8\times {10}^{21}$ cm^−3^, while the 800 nm wavelength light is reflected at the lower plasma densities when $\sim 1.7\times {10}^{21}$ cm^−3^ is reached. A filament-like low spatial frequency pattern (Fig. 4) is recognisable along the pulse propagation. Due to the FFT filtering horizontally extended fringes are apparent in the ratio images which are not pronounced in the originals (see, Fig. 3). As plasma density is increasing after laser pulse absorption, less of 400 nm light passes through. The initial stages of absorption and plasma formation show strong amplitude modulation on the time scales comparable with pulse duration of 45 fs. Kerr self-focusing at the very beginning of the transient is recognisable for the larger pulse energies (also evidenced in Fig. 5).

Transients of the phase changes at the focal point (Fig. 4) are shown in Fig. 5. Positive phase changes correspond to a decrease of the refractive index and vice versa. No permanent glass modifications were optically recognisable when the pump pulse energy was smaller than ~20 nJ. Almost twice larger phase changes were observed for the 400 nm imaging.

The void formation was observed by optical transmission for the pulses with energy >300 nJ/pulse^8^. In the case of 800 nm probe, there was a fast decrease of phase values, i.e., an increase in refractive index, +Δ*n*, at the end of the pulse at approximately 250 fs time moment. Thermalisation between electrons and ions/atoms of the glass matrix has not been finished at that time and this abrupt change is not related to the matrix temperature changes. It can be understood as fast removal of free carriers, i.e., a self-trapping of excitons which leads to defect formation at the much longer time scales (marked by the arrow in Fig. 5). Self-trapping is a well established in pure silica glasses where propensity of defect formation after band-to-band excitation is large.

The mechanism of self-trapping of exciton is generic for the wide bandgap dielectrics (the absorption edge of the B270 glass is 300 nm). Feasibility of self-trapping in B270 glass is partly supported by observed similarity of the breakdown and void formation in the wide silicate glass family which was distinct from the other glass forming phosphates and borates^26^. The non-bridging oxygen hole center (NBOHC) is a typical paramagnetic defect in silicate glass matrix which also has luminescence at around 650 nm wavelength (~1.9 eV) and is linked to optical damage precursor^27^. This dynamic process ~100 fs is comparable with pulse duration. The probe pulse energy is only *E*_pr_ = 10 nJ and its polarisation is perpendicular to the pump, hence, a coherent crosstalk was absent or small due to depolarisation. The phase contrast after long delays is determined by the mass density changes and absorbtion bands of defects which can be recalculated into refractive index changes via the Kramers-Kronig formalism.

In the case of 400 nm probe, there is no fast decrease of the phase values at the end of the pulse. At this high photon energy a free carrier absorption can be the major reason for considerably larger values of the phase (−Δ*n*); the pulse energy at 400 nm probe was *E*_pr_ = 10 nJ. A recognisable increase in phase value towards 1 ps and longer times is related to heating of the matrix which brings a decrease of refractive index, −Δ*n*; here we consider that strongly localised heating is expanding the matrix, hence, local density decreases. There is no apparent reduction of phase values upon formation of self-trapped exciton under 400 nm probing. It is plausible to assume that potential barrier for the trapped exciton is larger than 1.5 eV or 800 nm wavelength. It is noteworthy that NBOHC center excitation has a resonant absorption and the 400 nm wavelength could be out of the absorbance band.

**Figure 6:**
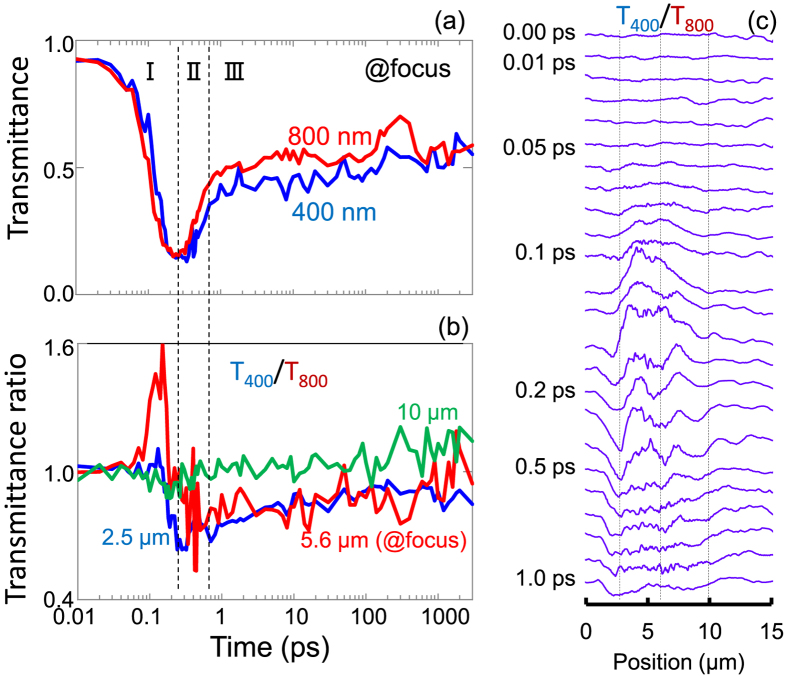
(**a**) Transmittance transient along propagation at 400 and 800 nm at the focal location (or 5.6 *μ*m axial coordinate). (**b**) Transmittance ratio *T*_400_/*T*_800_ at the different axial locations before and after focus. (**c**) Axial cross sections at different times at focus; central vertical line-marker shows location of the focus at low pulse energy. Pump pulse energy *E*_*p*_ = 200 nJ.

Figure 6 shows transmissivity ratios at different locations along propagation of the pulse; the focus is at *z* = 5.6 *μ*m. It is possible to define three regions in time evolution of the differential transmission through the different locations along the pulse propagation. The region I shows processes occurring during the pulse, the region II is where a fast evolution of recombination and electron movement before thermalisation takes place, and the region III captures consequence of thermalisation between electrons and host matrix.

The largest changes in transmissivity ratio *T*_400_/*T*_800_ are observed in the region I during the pulse. This is understandable due to large plasma densities exceeding 10^20^ cm^−3^ where short wavelength light has a higher transmission through the plasma at the focal region (Fig. 6(a)). The transmissivity ratio is almost unaccented before and after the focal region along pulse propagation, which indicates that fillamentation and self-focusing was not strong (Fig. 6(b)). There were on-axis changes in time observed in the phase and amplitude images in side-views^8^, which are well discernible in *T*_400_/*T*_800_ ratio (Fig. 6(c)). Most probably, it is related to the plasma density changes during pulse propagation and self-action of the pulse, i.e., once plasma is excited a back reflection of light from that region creates a stronger absorption ahead of the incoming pulse. At the geometrical focus in the region II, the ratio of transmittances is slightly below one and has strongest uncertainty. The region III (long delays), a plateau is observed. In this region light scattering dominates which has a strong size and wavelength dependence $\propto {r}^{6}/{\lambda }^{4}$ and affects 400 nm probe significantly (see discussion in the next section).

## Discussion and Outlook

The proposed method of two-color experiment could be used to elucidate fast events in plasma evolution and decay. How optically induced defects are evolving after plasma decay and the role of self-trapping of excitons needs better understanding. The self-trapped excitons are expected to form NBOHC which can reach high ~10^19^ cm^−3^ densities^28^. The NBOHC absorbs at 650 nm in silica glasses. Precursors of defects in glasses and wide bandgap materials^27^ can be investigated by the proposed pump-probe method.

Plasma regions with nanoscale cross sections can be considered as plasmonic nanoparticles whose extinction cross section - the total losses measured in transmission - are due to absorption and scattering contributions: ${\sigma }_{ext}\equiv {\sigma }_{abs}+{\sigma }_{sc}\propto {r}^{3}/\lambda +{r}^{6}/{\lambda }^{4}$ at the wavelength *λ* for the nanoparticle of radius, *r*. For example, a 100-nm-diameter gold particle has equal absorption and scattering cross sections with the latter dominating with increasing size^29^. Similar scaling is expected for the metal-like plasma. The size and concentration dynamics of the plasma regions are expected to bring about complex transients of transmissivity which are absorption, scattering, and diffraction dependent. The strong dependence of scattering on the geometrical size of plasma and wavelength allows to use this method in investigations of 3D nano-/micro-plasmas important in high-pressure high-density research^13^, especially suitable for a side-view imaging of axially extended focal regions of Bessel-Gaussian pulses^30–32^. An interesting extension of this side-view imaging method can be made by implementing the four-polarisation method^33^ for measurement of orientational anisotropy in the sample.

The proposed here high spatial resolution side-view imaging can find use in stimulated emission depletion STED-inspired fs-laser fabrication where de-excitation doughnut beam can localise inter-system crossing into the triplet or defect state down to nanoscale localisation onto optical axis^34^. The shown here self-trapping of exciton in glass could potentially be controlled by STED geometry. This is expected to help a nanoscale on-demand writing of defects for optical functions, the technique currently not available for deep-sub-wavelength resolution.

The proposed technique could be applied for two pulses of closely matching wavelengths (in order to probe the same refractive index) with separated enough fringes on the FFT image. This would provide a direct access to temporal evolution of the refractive index $(n+ik)$ at the focal region with high temporal resolution and imaging capability.

## Conclusions

We show a novel two-color probing method suitable to analyse fast processes induced by a pump pulse in a side-view imaging geometry. Spatial resolution was set the same by numerical filtering and was ~0.9 *μ*m for 400 and 800 nm light. Holographic numerical refocusing was implemented to compare side view pump-probe images at different delay times and to use FFT image processing. A possible explanation of a fast decrease of the FFT phase at the end of the pulse for 800 nm wavelength is an electron trapping which leads to defect formation. The transmissivity ratio at different wavelengths allows to interrogate the opaque regions of plasma, i.e., provides tool to characterise plasmas of different densities.
